# Efferent Control of the Electrical and Mechanical Properties of Hair Cells in the Bullfrog's Sacculus

**DOI:** 10.1371/journal.pone.0013777

**Published:** 2010-10-29

**Authors:** Manuel Castellano-Muñoz, Samuel H. Israel, A. J. Hudspeth

**Affiliations:** Howard Hughes Medical Institute and Laboratory of Sensory Neuroscience, The Rockefeller University, New York, New York, United States of America; Harvard University, United States of America

## Abstract

**Background:**

Hair cells in the auditory, vestibular, and lateral-line systems respond to mechanical stimulation and transmit information to afferent nerve fibers. The sensitivity of mechanoelectrical transduction is modulated by the efferent pathway, whose activity usually reduces the responsiveness of hair cells. The basis of this effect remains unknown.

**Methodology and Principal Findings:**

We employed immunocytological, electrophysiological, and micromechanical approaches to characterize the anatomy of efferent innervation and the effect of efferent activity on the electrical and mechanical properties of hair cells in the bullfrog's sacculus. We found that efferent fibers form extensive synaptic terminals on all macular and extramacular hair cells. Macular hair cells expressing the Ca^2+^-buffering protein calretinin contain half as many synaptic ribbons and are innervated by twice as many efferent terminals as calretinin-negative hair cells. Efferent activity elicits inhibitory postsynaptic potentials in hair cells and thus inhibits their electrical resonance. In hair cells that exhibit spiking activity, efferent stimulation suppresses the generation of action potentials. Finally, efferent activity triggers a displacement of the hair bundle's resting position.

**Conclusions and Significance:**

The hair cells of the bullfrog's sacculus receive a rich efferent innervation with the heaviest projection to calretinin-containing cells. Stimulation of efferent axons desensitizes the hair cells and suppresses their spiking activity. Although efferent activation influences mechanoelectrical transduction, the mechanical effects on hair bundles are inconsistent.

## Introduction

Hair cells in the acousticolateralis system are responsible for the detection of sound, acceleration, vibration, and water flow. Both the high sensitivity and the sharp frequency selectivity in these cells rely upon an active process that supplies energy to the hair bundle, the hair cell's mechanosensitive organelle. Mechanical stimuli detected by the mechanoelectrical-transduction channels in the bundle evoke receptor potentials that trigger the release of neurotransmitter at the hair cell's ribbon synapses and thus stimulate afferent fibers.

The sacculus is a vestibular organ that in various species is sensitive to head tilt, seismic vibrations, and airborne sound. Saccular hair cells are extensively innervated by afferent fibers whose cell bodies are localized in peripheral ganglia [1], [2]. Vestibular hair cells are also directly innervated by efferent fibers that originate in brainstem nuclei [3]. Efferent activation generally reduces the sensitivity of vestibular and auditory hair cells to mechanical stimulation. The functional consequences of this inhibition include reduction in the sensitivity to undesired ambient sounds, cancellation of self-stimulation during motor activity, improvement in the signal-to-noise ratio, protection from overstimulation, and increased temporal resolution in exchange for diminished frequency tuning [4], [5].

Auditory and vestibular efferents play a role in mechanotransduction by regulating the membrane potential of hair cells and afferent terminals. In addition, mammalian cochlear efferents modulate the membrane potential of outer hair cells to regulate the active process by controlling electromotility [4]. Because electromotility is unique to cochlear outer hair cells in mammals, however, it is unlikely that there is a unified mechanism by which efferent stimulation exerts a mechanical effect on the active process. In fact, the effects of efferent activity on the mechanical properties of the hair bundle have not been investigated under physiological conditions.

Using the bullfrog's sacculus as a model system, we studied the effect of efferent stimulation on the activity of hair cells. We used immunocytochemistry to characterize the distribution of efferent terminals and ribbon synapses. By electrophysiological recordings, we investigated the effect of efferent activity on the electrical properties of hair cells. Finally, we used photomicrometry to measure the mechanical effect of efferent activation on hair-bundle displacement.

## Materials and Methods

### Experimental preparation

After approval of research protocol 04044 by the Institutional Animal Care and Use Committee of The Rockefeller University, bullfrogs (*Rana catesbeiana*) were anesthetized by immersion in ice, pithed, and decapitated. Sacculi were dissected in oxygenated standard saline solution containing 110 mM Na^+^, 2 mM K^+^, 2 mM Ca^2+^, 118 mM Cl^−^, 3 mM **D**-glucose, and 5 mM HEPES. For immunocytochemistry, the eighth cranial nerve was cut close to the macula, or sensory epithelium. For physiological recordings, the nerve was instead severed at the level of the brainstem and the utricular branch, leaving the contact with the sensory epithelium intact. The cut end of the nerve was then pulled into a suction electrode made of silicone rubber. The saccular macula and the surrounding epithelium, together with the eighth cranial nerve, were glued over a 1-mm-diameter hole in a plastic coverslip with tissue-compatible acrylic adhesive (Iso-dent, Ellman International, Hewlett, NY). The preparation was then mounted on a two-compartment chamber to expose its apical and basal surfaces to their native physiological conditions. While the basolateral surfaces of the hair cells were bathed in standard saline solution, their hair bundles were immersed in artificial endolymph containing 2 mM Na^+^, 118 mM K^+^, 0.25 mM Ca^2+^, 118 mM Cl^−^, 3 mM **D**-glucose, and 5 mM HEPES. After exposing the apical surfaces of the hair cells to endolymph supplemented with 67 µg/ml subtilisin (protease type XXIV; Sigma, St. Louis, MO) for 25 min or 100 µg/ml collagenase IV (Worthington, Lakewood, NJ) for 10 min at room temperature, we removed the otolithic membrane to expose the hair bundles. Electrophysiological and micromechanical recordings were restricted to the large, cylindrical, central hair cells of the sacculus [6]. At the end of some experiments, the upper compartment was filled with a solution of 50 µM Alexa 568 to check for possible leakage between compartments. All solutions had a pH of 7.3 and an osmolarity of 225–245 mmol/kg. The dissections and experiments were performed at room temperature.

### Antibody generation

A conserved sequence in the N-terminal region of synapsin I (Fig. S1A) was used to synthesize the peptide CYLRRRLSDSNFMANLPNGY (Proteomics Resource Center, Rockefeller University, NY), which was conjugated to keyhole limpet hemocyanin through the added N-terminal cysteine and was injected into two rabbits, one guinea pig, and one chicken by standard protocols (Cocalico Biologicals Inc., Reamstown, PA). The polyclonal antisera Rb1497, Rb1498, Gp118, and Ch13 were purified from immune serum by binding to antigen (SulfoLink Immobilization Kit, Pierce ThermoScietific, Rockford, IL), dialyzed (Slide-A-Lyzer 10K, Pierce, Rockford, IL), and concentrated (Vivaspin, GE Healthcare, Chalfont St. Giles, UK). Immunofluorescence results were equivalent with the four antisera.

### Immunofluorescence labeling

Isolated sacculi were incubated for 1 h at 4°C in phosphate buffered saline (PBS) solution containing 4% formaldehyde and 0.1% Triton X-100. After washing four times for 10 min each at room temperature in PBS containing 0.1% Triton X-100, we incubated the sacculi for 30 min in the same solution supplemented with 5% bovine serum albumin (BSA). The specimens were then incubated with primary antisera at a dilution of 1∶100-1∶1000 in PBS containing 0.1% Triton X-100 and 5% BSA for 2 h at room temperature or overnight at 4°C. The antisera included those against calretinin (polyclonal, AB1550, Chemicon), CtBP2 (polyclonal, #1869), parvalbumin 3 (polyclonal, #799), SV2 (monoclonal, DSHB), alpha-tubulin (monoclonal, 6G7, Sigma), and beta-actin (monoclonal, A5441, Sigma). After washing five times for 10 min each at room temperature, we incubated the samples with Alexa 488/568-conjugated secondary antibodies (Invitrogen, Carlsbad, CA) at a dilution of 1/500. Where mentioned, DAPI or Alexa 568-conjugated phalloidin was added during the incubation with the secondary antibodies. After washing five times for 10 min each at room temperature, we mounted the specimens between two coverslips with Vectashield (Vector Laboratories, Burlingame, CA).

### Immunoblotting

Tissues were homogenized in PBS containing 0.1% SDS, 1% Triton X-100, 2 mM EDTA, 1 mM PMSF, and protease inhibitors (Roche, Hoffmann-La Roche, NJ), centrifugued at 10,000×g at 4°C for 5 min in a QIAshredder Spin Column (Qiagen Inc., Valencia, CA), diluted with NuPAGE LDS sample buffer and NuPAGE reducing agent (Invitrogen Corp., Carlsbad, CA), incubated at 100°C for 5 min, loaded on NuPAGE Novex 10% Bis-Tris gels, and transferred onto PVDF membranes. Blots were incubated with primary antibodies overnight at 4°C and probed by immunoblotting with horseradish peroxidase-labeled secondary antibodies and enhanced chemiluminescence detection (ECL Plus, GE Healthcare, Chalfont St. Giles, UK). In the preadsorption experiments, antibody binding was blocked by preincubation for 5 h at 4°C with a 20-fold molar excess of the peptide.

### Electron microscopy

Specimens were prepared for transmission electron microscopy by published techniques [7].

### Microelectrode recording

Sharp electrodes were produced on a horizontal electrode puller (P-2000; Sutter Instrument, Novato, CA) from borosilicate glass capillaries (1B120F-3, World Precision Instruments, Sarasota, FL). To facilitate penetration of the apical surface of a hair cell, the tapered end of each microelectrode was inserted horizontally into a drop of water and bent 0.5 mm from the tip by heating the drop close to the insertion point [8]. Filled with 3 M potassium acetate and 75 mM KCl, the electrodes displayed resistances of 50–150 MΩ. Each electrode's tip was inserted into the apical surface of a hair cell with a micromanipulator (ROE-200, Sutter Instrument, Novato, CA). Recordings were made with a current-clamp amplifier (Axoclamp-2B, Axon Instruments Inc., Burlingame, CA). All recordings were low-pass filtered at 3–5 kHz with an eight-pole Bessel filter (Wavetek 852, San Diego, CA) and sampled at 50-µs intervals. Equivalent results were obtained by peeling off the otolithic membrane with an eyelash with or without protease treatment or by passing the electrode through the cavity in the otolithic membrane above each hair cell.

### Microscopic apparatus

Experiments were conducted under a BX51WI upright microscope (Olympus, Center Valley, PA) equipped with a 200-W mercury lamp (Excite exacte, EXFO, Mississauga, Ontario, Canada), a bandpass filter (HQ600/200m-2p, Chroma, Rockingham, VT), and standard differential-interference-contrast optics. The image formed by a 40X water-immersion objective lens of numerical aperture 0.8 and working distance 3.3 mm was either projected onto a CCD camera (WAT-660D, Watec, Orangeburg, NY) or viewed directly through the eyepieces.

### Force-fiber fabrication

The techniques for mechanical stimulation, imaging, and optical calibration have been described in detail elsewhere [9]. To measure the motion of a hair bundle, the tip of a glass fiber about 300 µm in length and 1 µm in diameter was attached to the kinociliary bulb of the hair cell and used both to apply force and to report bundle movements. To improve contrast and adhesion, the fiber was sputter-coated with gold-palladium (Anatech Hummer, Union City, CA) and its tip immersed in 1 mg/ml concanavalin A (type VI, Sigma, St. Louis, MO) prior to the experiment. The fibers had stiffnesses of 6-126 µN⋅m^−1^ and drag coefficients of 21–180 nN⋅s⋅m^−1^ as determined by calculation from the power spectra of the Brownian motion of their tips in water.

### Photodiode micrometry

After removal of the differential-interference-contrast components and the microscope's frosted-glass screen, the image of the fiber tip was magnified 958X and projected onto a dual photodiode (SPOT-2D OSI Optoelectronics, Hawthorne, CA). A custom-made circuit attached to the photodiode provided current-to-voltage conversion. The photodiode and circuit were mounted on a two-axis linear stage to allow centering of the shadow of the fiber's tip between the two photosensitive cells. Prior to reaching the photodiode, the magnified image was reflected from a 45° mirror mounted on a piezoelectric actuator (P-840.60, Physik Instrumente, Auburn, MA). To calibrate the photodiode without moving the fiber, a series of displacement steps of alternating polarity was delivered to the piezoelectric actuator, yielding a linear relationship between the probe's displacement and the photomicrometer's output voltage. Hysteresis in the piezoelectric actuator was compensated by delivering a 65%-overshooting prepulse before each calibration step. Recordings used the piezoelectric actuator to deliver a calibration prepulse equivalent to 20 nm at the level of the fiber tip. The photodiode system was in turn calibrated with a heterodyne interferometer (OFV3001/501, Polytec GmbH, Waldbronn, Germany). The fiber was secured at its base to a second stack-type piezoelectric actuator (P835.10; Physik Instrumente, Auburn, MA) driven by a matched power supply (E-663.00, Physik Instrumente, Auburn, MA). The piezoelectric actuator was mounted on an electrophysiological micromanipulator (ROE-200, Sutter Instrument, Novato, CA) and was calibrated with the interferometer.

### Data collection and analysis

All devices were controlled by custom-written software (LabVIEW 7.0, National Instruments, Austin, TX). Multifunctional data acquisition boards (PCI-6120, PCI-6733, National Instruments) were used for analog device control and data acquisition. Data analysis was performed with custom-written routines in Matlab (The Mathworks, Natick, MA). Postsynaptic potentials were analyzed with MINIANALYSIS (SYNAPTOSOFT; Jaejin Software, Leonia, NJ). Graphical data were analyzed with ImageJ (NIH).

## Results

### Efferent innervation of the bullfrog's sacculus

Although the sacculus receives dense efferent innervation [10], [11], the distribution of efferent synaptic terminals is unknown. In order to characterize the anatomical distribution of efferent presynaptic terminals, we analyzed the expression pattern of synapsin I, a protein that is expressed in efferent terminals but not at ribbon synapses [12], [13]. Using a conserved region at the N-terminus as an antigen, we generated polyclonal antisera against synapsin I (Fig. S1A). Western-blot analysis with brain homogenates from the bullfrog, chicken, and mouse showed immunoreactive proteins of relative molecular masses near 55 kDa and 80 kDa (Fig. S1B) that likely correspond to different synapsin isoforms [14]. This signal was absent when preimmune serum was used or when antibodies were preadsorbed with 20-fold molar excess of the antigen by western-blot or immunofluorescence (Fig. S1B-C).

**Figure 1:**
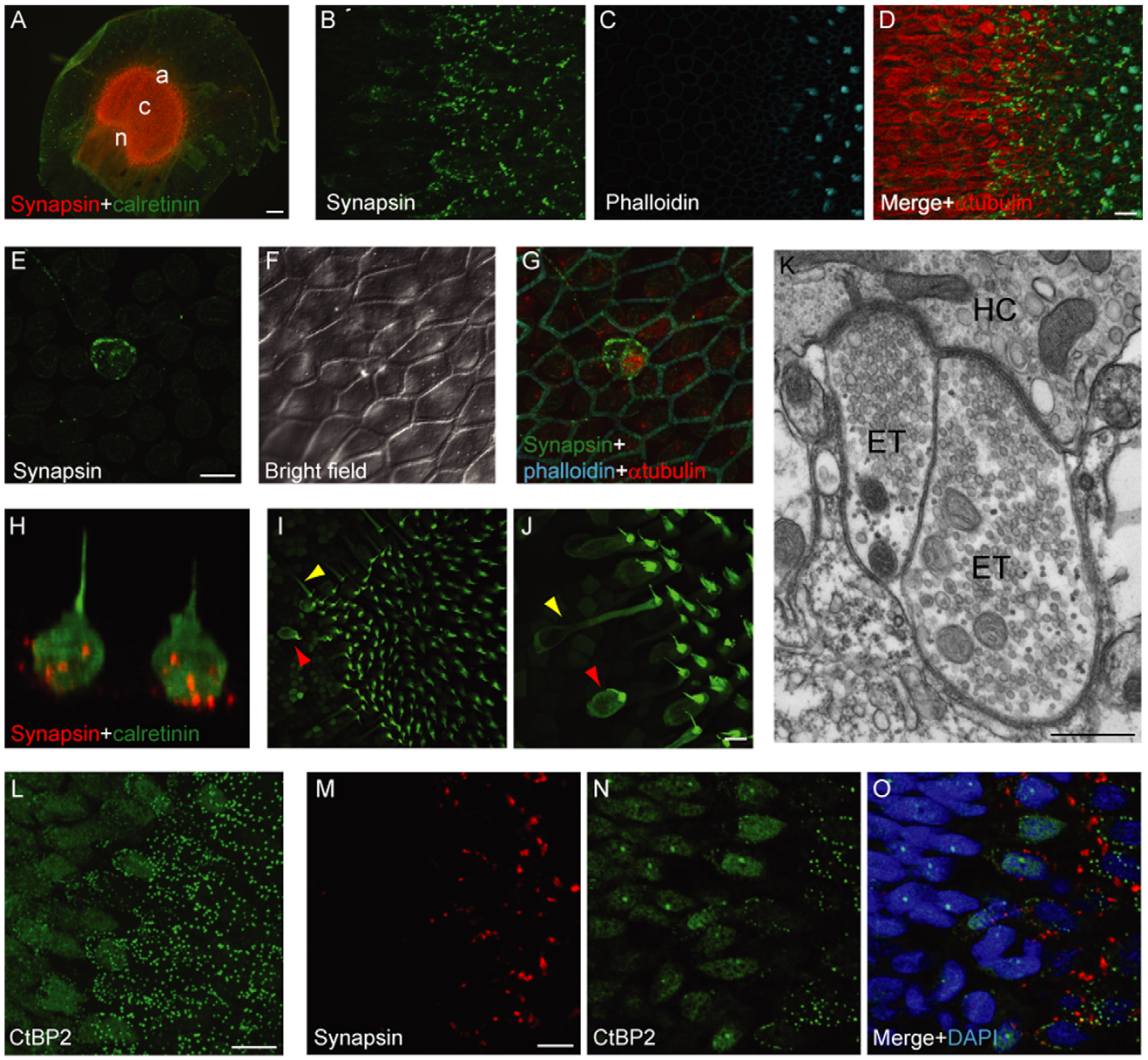
Efferent innervation of saccular hair cells. ***A***, A wholemount immunofluorescence micrograph shows that synapsin I is highly expressed throughout the saccular macula. The regions used in the statistical analysis are indicated: *n*, neural; *c*, central; *a*, abneural. ***B*** **–** ***D***, A higher-magnification stack of confocal images of the macular edge depicts efferent terminals labeled by an anti-synapsin I antiserum (Rb1497) exclusively within the macula, an area demarcated by phalloidin-labeled hair bundles. Note the high expression of alpha-tubulin in extramacular epithelial cells. ***E*** **–** ***G***, A maximal-intensity projection of confocal *Z*-stacks portrays an extramacular hair cell innervated by efferent terminals. ***H***, A three-dimensional confocal reconstruction shows two nearby extramacular hair cells marked by antisera against calretinin (green) and synapsin I (red). ***I*** **–** ***J***, A *Z*-stack projection shows the morphological variability of calretinin-positive hair cells in the macular periphery. Note the occurrence of cylindrical (red arrowheads) and flask-shaped cells (yellow arrowhead). ***K***, As visualized by transmission electron microscopy, two efferent terminals (ET) contact a single hair cell (HC) at adjacent efferent synapses. Note the abundance of synaptic vesicles in the presynaptic terminals and the postsynaptic cisterns in the hair cell. ***L***, A *Z*-stack projection of the macular periphery shows that an antiserum against CtBP2 labels synaptic ribbons in macular hair cells and nuclei in the epithelial cells outside the macula. ***M*** **–** ***O***, A confocal section in the macular periphery shows the abundance of efferent and afferent terminals labeled by respectively an antiserum against synapsin I (Gp118) and one directed against CtBP2. Scale bars: ***A***, 100 µm; ***D***, ***E***, ***J***, ***L***, ***M***, 10 µm; ***K***, 500 nm.

We used standard epifluorescence and confocal microscopy to analyze the expression pattern of synapsin I in the saccular epithelium of the bullfrog. Efferent terminals were distributed throughout the macula with no obvious spatial pattern (Fig. 1A–D). Synapsin I colocalized with SV2 (data not shown), another synaptic-vesicle protein present at efferent terminals [15]. Synapsin I labeling was dense at the base of each hair cell (Fig. 1H and Video S1) but only sporadic apical to the nucleus. Each macular hair cell displayed an average of 10±5 efferent terminals (mean ± standard deviation, *n* = 311 cells). Extramacular hair cells, whose function is unknown, were also highly innervated by efferent terminals (Fig. 1E–H and Video S2).

On the basis of morphology, protein expression, and electrophysiological properties, the hair cells of the frog's sacculus may be classified into at least three classes [2], [6]. The first group consists of cylindrical cells with large voltage-dependent Ca^2+^ currents (Fig. 1I–J). The second class includes flask-shaped cells that display large Ca^2+^-dependent K^+^ currents and express the Ca^2+^ buffer calretinin [16], [17]. The third group includes calretinin-positive cells with variable morphology that are localized exclusively along the perimeter of the macula and likely represent newly formed cells [18]. To determine whether these three types of cells are differentially innervated by the efferent pathway, we quantified the efferent terminals at three different positions across the macula (Fig. 1A). We observed a higher occurrence of efferent terminals along the macular perimeter with no difference between the neural and abneural edges. Furthermore, hair cells expressing calretinin displayed significantly more efferent contacts in all regions examined (Table 1).

**Table 1:** Number of efferent synaptic terminals and synaptic ribbons in macular hair cells.

Hair-cell group	Number of efferent terminals		Number of synaptic ribbons	
Abneural CR-	7.22±0.44	(23)	34.67±1.99*	(24)
Abneural CR+	14.83±0.63	(48)	37.35±1.38*	(46)
Central CR-	6.09±0.21	(76)	20.67±0.51	(75)
Central CR+	9.61±0.37	(36)	8.46±0.85	(28)
Neural CR-	7.22±0.36	(41)	32.63±1.31*	(38)
Neural CR+	13.99±0.56	(87)	31.56±1.22*	(54)
Extramacular	9.03±0.66	(31)	n.d.	

Values are means ± SEMs. For each group, the number of hair cells tested is given in parentheses. Calretinin-positive hair cells (CR+) and calretinin-negative hair cells (CR–) were statistically analyzed using a two-tailed Student's *t*-test with unequal variance. All pairwise comparisons were significant at *p*<10^−12^ except for the two indicated with asterisks, which were not significant at *p*<0.5. Efferent synaptic terminals were identified as synapsin-positive structures surrounding the basolateral regions of hair cells. Synaptic ribbons were determined by the presence of distinct CtBP2-positive signals surrounding hair-cell nuclei. Z-stack confocal images with 0.3-µm increments were analyzed in a total of 576 hair cells from six different sacculi.

To explore the fine structure of efferent presynaptic terminals in hair cells, we performed electron microscopy on the sacculus. In accordance with the typical pattern of efferent synapses [19], each presynaptic terminal contained a dense cluster of synaptic vesicles apposed to a moderate presynaptic density. The associated hair cell bore a modest postsynaptic density with a postsynaptic cisterna in the adjacent cytoplasm (Fig. 1K), confirming the ultrastructural efferent morphology shared with other preparations.

Although saccular hair cells are innervated by both afferent and efferent fibers, little is known about the relative distributions of these two synaptic classes. We assayed the number of synaptic ribbons in the three types of hair cells by labeling with a polyclonal antiserum directed against C-terminal binding protein 2 (CtBP2). This antiserum labeled the synaptic ribbons in all macular hair cells, whereas it labeled the nuclei of epithelial cells outside the macula (Fig. 1L–O and Video S3). At the center of the macula, the flask-shaped, calretinin-positive cells contained a significantly smaller number of synaptic ribbons than did the cylindrical hair cells (Table 1). Regardless of their calretinin expression, the hair cells at the perimeter of the macula displayed more synaptic ribbons than did those at the center of the macula. We unexpectedly observed no synaptic ribbons in the extramacular hair cells. There was no evident spatial relationship between afferent and efferent terminals.

Taken together, these results demonstrate that all hair cells in the bullfrog's sacculus, especially those expressing calretinin, are highly innervated by efferent fibers. This pattern suggests a fundamental role for efferent control in the responsiveness of saccular hair cells.

### Efferent modulation of the hair-cell resting potential

The electrical properties of hair cells are known to be modulated by the efferent pathway in a variety of sensory receptors [5]. In the frog's sacculus [20], [21] as well as in the cochleas of turtles [22] and rats [23], efferent stimulation produces inhibitory postsynaptic potentials owing to the release of acetylcholine.

**Figure 2:**
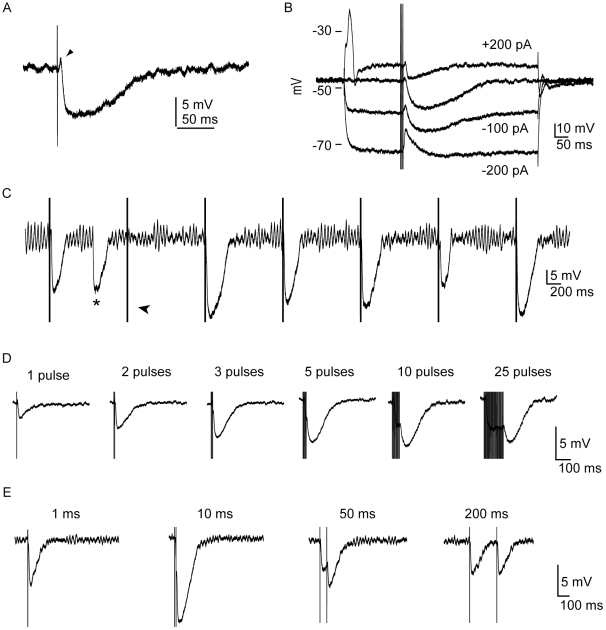
Electrophysiological effect of efferent stimulation. ***A***, A single shock of the efferent innervation triggered a brief depolarization of a hair cell (arrowhead) followed by long-lasting hyperpolarization (single trace; one compartment, 4 mM Ca^2+^, resting potential −57 mV). In this and subsequent panels, the stimulus artifact has not been removed. ***B***, The depolarizing component of the response as well as reversed potassium current flow were evident at membrane potentials negative to the equilibrium potential for K^+^ (single trace; one compartment, 4 mM Ca^2+^, resting potential −48 mV). **C**, The electrically evoked inhibitory postsynaptic potentials, which sometimes failed (arrowhead), resembled those obtained spontaneously (asterisk; resting potential −49 mV). ***D***, The magnitude of the inhibitory postsynaptic potential was increased by raising the number of stimulus pulses up to a maximum of five (single traces; one compartment, 4 mM Ca^2+^). ***E***, The amplitude of the inhibitory postsynaptic potential after pairs of efferent shocks peaked at a separation of 10 ms.

To study the role of efferent activation on saccular hair-cell receptor potential, we first characterized its effect on the resting potential. We used a suction electrode to stimulate the efferent nerve bundle while recording intracellularly from hair cells. Upon efferent stimulation, the majority of hair cells displayed a response with two components, a brief depolarization followed by a strong, long-lasting hyperpolarization (Fig. 2A). This response is consistent with an inward Ca^2+^ current through cholinergic receptors and a subsequent outward K^+^ current through SK2 channels [22]. The prominent hyperpolarization reversed in sign between −75 mV and −100 mV (Fig. 2B), close to the reversal potential for K^+^, suggesting a direct role for K^+^ currents in this component of the response [21]. Consistent with previous reports [23], [24], spontaneous efferent-like activity was occasionally observed (Fig. 2C).

Inhibitory postsynaptic potentials were evoked by 0.2-mA stimuli 0.05–3 ms in duration. Electrical stimuli of greater amplitude or duration did not produce larger effects. Responses were observed in a total of 52 hair cells, with a maximum hyperpolarization of 27 mV and an average value in 4 mM-Ca^2+^ saline of 6.4±4.3 mV. In six cells that displayed inhibitory postsynaptic potentials in the presence of 2 mM Ca^2+^, the hyperpolarization averaged 4.0±2.4 mV. Within the same cells, the responses triggered by efferent stimulation also varied in amplitude and length from trial to trial (Figures 2C). The time course of repolarization was amplitude-dependent (Fig. S2A). The fact that inhibitory postsynaptic potentials do not scale simply, in contrast to the behavior of afferent excitatory postsynaptic potentials [25], likely reflects non-exponential processes associated with Ca^2+^ buffering [26], [27].

Single electrical stimuli delivered to efferent axons generally produced small, sporadic responses. Rapid, periodic trains of electrical stimuli delivered at a frequency near 200 Hz increased the probability and amplitudes of inhibitory postsynaptic potentials (Fig. 2D and Table 2). The amplitude peaked when trains of roughly five current pulses were delivered. Although longer trains did not evoke larger responses, they increased the duration of the hyperpolarizing component (Fig. 2D).

**Table 2:** Dependence of IPSP magnitude on the number of efferent stimuli.

Number of pulses	*n*	*p*	Amplitude (mV)	Rise time (ms)	Decay time (ms)
1	64/150	0.43±0.04	1.6±0.4	9±9	31±19
2	115/150	0.77±0.03	2.0±0.5	8±5	39±17
4	146/150	0.97±0.01	2.2±0.5	13±6	47±16
5	148/150	0.99±0.01	2.6±0.4	22±8	59±16

Each line shows the number of IPSPs (*n*), the estimated event probability (*p*) with its standard deviation, and the mean with its standard deviation for three IPSP parameters after 150 trials. IPSPs were recorded in the same cell and triggered by 0.05-ms stimulus pulses separated by 5 ms.

The synaptic effect additionally depended on the delay between efferent current pulses, with a maximal response evoked by stimuli delivered 3–10 ms apart (Fig. 2E and Fig. S2B). This result accords with that for cochlear hair cells [23], [28], suggesting similarities in the efferent physiology of the auditory and vestibular systems.

### Efferent modulation of the hair-cell receptor potential

In the turtle's cochlea, efferent activity transforms sharply tuned electrical resonance into overdamped relaxation [22]. The consequences of efferent stimulation on the electrical resonance of vestibular hair cells, however, have not been well characterized. Using a two-compartment apparatus to mimic the ionic conditions *in vivo*, we characterized the electrical responses of hair cells to injected current in control conditions and during stimulation of the efferent pathway.

**Figure 3:**
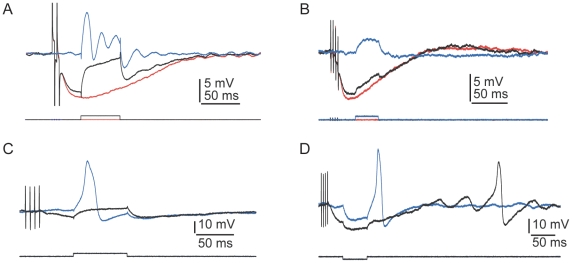
Physiological characteristics of hair cells and electrophysiological effects of efferent stimulation. ***A***, In response to a depolarizing current pulse (lower trace), electrical resonance (blue trace) was eliminated by a preceding train of efferent stimuli (black trace). The red trace shows the postsynaptic potential in the absence of a current pulse (average of ten presentations, one compartment, 4 mM-Ca^2+^ saline, resting potential −57 mV). ***B***, In response to a depolarizing current pulse (lower trace), the membrane resistance was reduced by a preceding train of efferent stimuli (black upper trace). The blue trace depicts the control situation with no efferent stimulation; the red trace shows the control situation with no depolarizing current pulse (one compartment, 4 mM-Ca^2+^ saline, resting potential −53 mV). ***C***, A preceding train of efferent stimulation prevented the occurrence of an action potential in response to a depolarizing current pulse (one compartment, 4 mM-Ca^2+^ saline, resting potential −57 mV). ***D***, Efferent stimulation blocked rebound spikes and modulated the timing of spontaneous oscillation in the membrane potential (one compartment, 4 mM-Ca^2+^ saline, resting potential −50 mV).

In the absence of efferent stimulation, we found 75 cells that displayed electrical resonance that could be observed either as damped oscillations in response to depolarizing current steps (blue trace in Fig. 3A) or as increases in membrane-potential oscillation at particular frequencies in response to frequency sweeps (data not shown). We also encountered 60 hair cells that did not remain in a subthreshold regime in response to applied currents, but responded to depolarizing current steps with broad action potentials (blue trace in Fig. 3C). The slow timecourses of these spikes suggested that they were Ca^2+^-based action potentials, which have been reported in a similar preparation in response to mechanical stimulation [29]. The spike threshold varied between cells; the smallest current that elicited a spike was 5 pA. The maximum amplitude of the spikes was approximately 40 mV. These action potentials, which could be generated individually or in trains (Fig. S2C-D), were occasionally spontaneous (Fig. S2E) and also arose as rebound spikes following hyperpolarizing current steps (Fig. S2D). The frequency of spike generation increased with the magnitude of current injection (Fig. S2C) and Ca^2+^ concentration (not shown). In addition to the spike activity, most of these cells also displayed electrical resonance in response to subthreshold excitation. We obtained action potentials using saline solutions containing 1 mM, 2 mM, or 4 mM Ca^2+^.

Following efferent stimulation, in addition to hyperpolarization of the resting potential, inhibitory postsynaptic potentials also prevented spontaneous oscillation of the membrane potential in highly tuned hair cells (Fig. 2C)[20]. After the inhibitory synaptic potential, the spontaneous oscillations of the membrane potential resumed. In 11 cells that presented robust tuning, efferent stimulation eliminated the typical damped oscillation in the membrane potential following depolarization (Fig. 3A). In eight hair cells lacking electrical resonance, the steady-state conductance increased at the peak of the synaptic potential relative to that at the resting potential (Fig. 3B and D), consistent with an increase in the activation of SK channels. The steady-state resistance in resonant and spiking cells, however, was variable and often difficult to quantify. In eight hair cells, efferent activity also blocked the appearance of action potentials after depolarization and anode-break stimulation (Fig. 3C and D).

Although it is generally accepted that the efferent systems of acousticolateralis organs are inhibitory in nature, there have been reports of efferent enhancement of vestibular afferent responses [30], [31], [32]. In the sacculus, only inhibitory modulation has been found [20], [21], [33], [34], [35], [36]. We recorded from three hair cells, however, in which efferent stimulation triggered an excitatory response. Similarly to the generation of rebound spikes following hyperpolarizing current steps, the repolarizing phase of the inhibitory postsynaptic potential triggered an action potential (Fig. S2F). This result indicates that the efferent pathway can play an excitatory role.

### Efferent effects on hair-bundle motion

In order to distill meaningful mechanical information from background noise, hair cells use an active process that amplifies and tunes their inputs. Ca^2+^ plays a key role in this active process, both by adjusting the hair bundle to a position of instability through slow adaptation, and by mediating the transduction-channel reclosure, or fast adaptation, that powers amplification [37].

We hypothesized that efferent activity might modulate hair-bundle motion by altering the concentration of Ca^2+^ in the stereocilia. On a slow timescale, efferent activation might act through the diffusion of Ca^2+^ from synaptoplasmic cisternae after Ca^2+^-induced Ca^2+^ release [38]. Alternatively, and on a faster timescale, the hyperpolarizing component of the efferent response might increase the Ca^2+^ flux through mechanoelectrical-transduction channels into stereocilia by augmenting the driving force on Ca^2+^.

**Figure 4:**
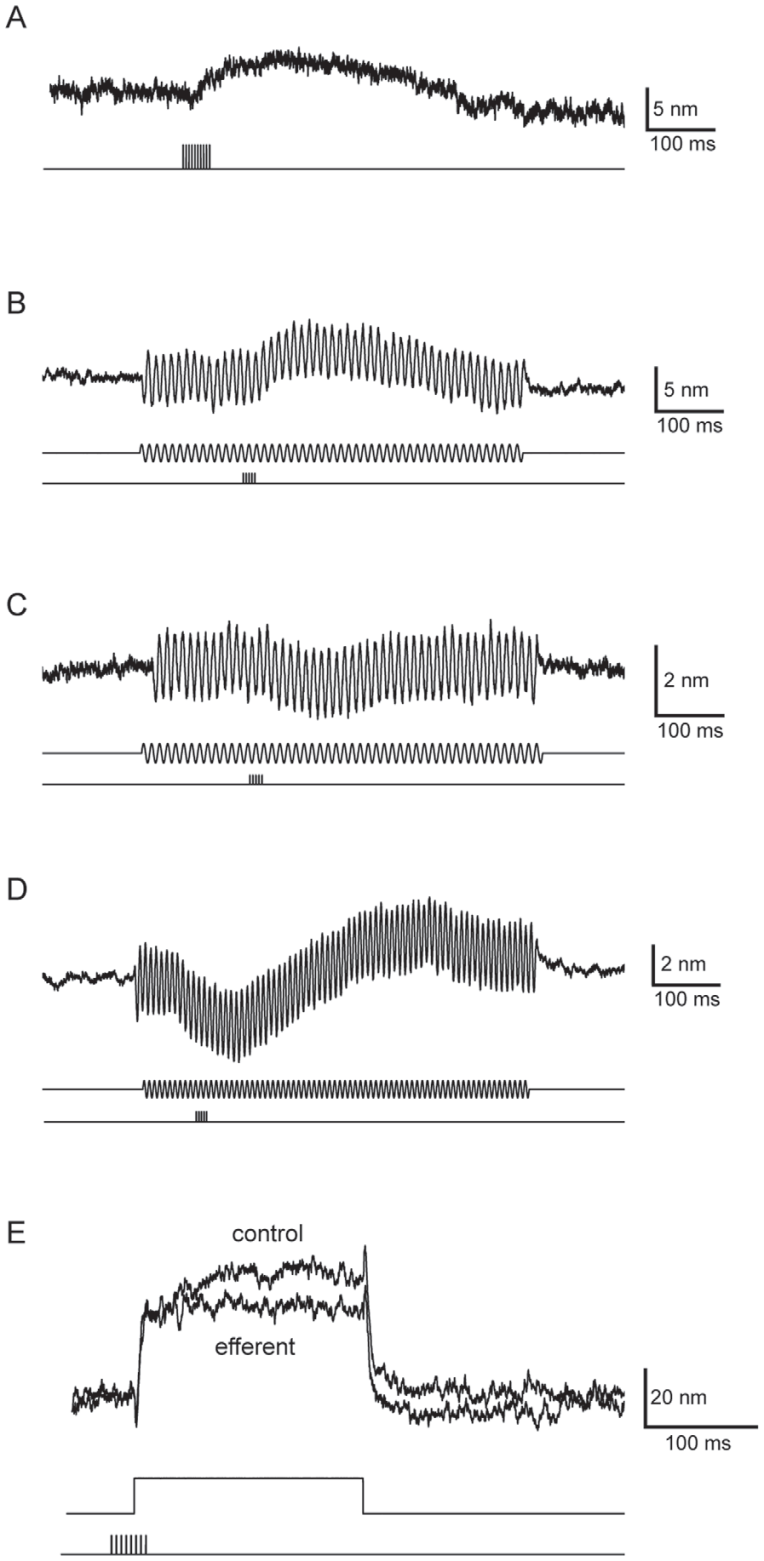
Effects of efferent stimulation on hair-bundle position. ***A***, A train of efferent stimuli (lower trace) triggered movement of the hair bundle attached to a compliant probe (upper trace; one compartment, 2 mM-Ca^2+^ saline). In this and subsequent traces, an upward deflection represents movement in the positive direction, toward the kinocilium. ***B***, During sinusoidal mechanical stimulation (middle traces), the same hair bundle as in ***A*** shifted in the positive direction after efferent stimulation without a change in the amplitude of the motion (one compartment, 2 mM-Ca^2+^ saline, ±30 nm). ***C***, In another hair cell, a similar paradigm induced a shift in the negative direction (two compartments, 0.25 mM-Ca^2+^ endolymph, 2 mM-Ca^2+^ saline, ±40 nm). ***D***, Efferent activity occasionally triggered a biphasic movement of the hair bundle (two compartments, 0.25 mM-Ca^2+^ endolymph, 2 mM-Ca^2+^ saline, ±40 nm). The traces represent the averages of 20–50 repetitions. ***E***, A hair bundle was stimulated by application of a 150-nm displacement pulse (middle trace) at the base of a compliant fiber. After displacement of the bundle toward its kinocilum, the bundle's movement (top trace) displayed slow adaptation before reaching a steady-state displacement of 44 nm. When the same protocol was preceded by efferent stimulation (bottom trace), the steady-state displacement dropped to 32 nm (two compartments, 0.25 mM-Ca^2+^ endolymph, 2 mM-Ca^2+^ saline, fiber stiffness 42 µN m^-1^, fiber drag coefficient 124 nN s m^-1^).

To assess these possibilities, we studied the effect of efferent stimulation on hair-bundle motion by recording the displacement of a compliant fiber attached to the hair bundle's tip. To mimic physiological ionic conditions, we filled the upper and lower compartments of the experimental chamber with endolymph and perilymph, respectively. Approximately 100 ms after the onset of a train of efferent stimuli, the resting position of the hair bundle shifted about 3.5 nm toward the kinocilium, then returned to its original position (Fig. 4A). The unidirectional bundle displacement following efferent stimulation never exceeded 5 nm. The duration of this displacement resembled but slightly exceeded that of the inhibitory postsynaptic potentials recorded in separate experiments. Although efferent stimulation did not appear qualitatively to affect the behavior of 36 spontaneously oscillating hair bundles, a shift in bundle position was occasionally found after averaging tens of repetitions.

To investigate the effect of efferent stimulation during mechanical stimulation, we monitored the motion of hair bundles in response to sinusoidal force. The resting point of the bundle shifted during efferent activity (Fig. 4B). Although we occasionally measured a variation in the amplitude of the bundle motion following efferent stimulation (Fig. S3), this effect was small and too inconsistent to be quantified. The amplitude of the evoked bundle motion did not change with the frequency of mechanical stimulation over the range from 4 Hz to 130 Hz. Protracted efferent stimulation by brief trains of stimulus pulses separated by short delays maintained the shifted bundle position. We obtained similar results whether we used a two-compartment experimental chamber to mimic physiological ionic conditions or employed a single chamber containing saline solution with 1.5 mM Ca^2+^. We consistently measured a shift in bundle position following efferent stimulation in a total of 28 hair cells. The direction of the displacement, however, was variable: 21 hair bundles moved in the positive direction, toward the kinocilium, whereas seven bundles moved in the opposite direction (Fig. 4B and C). In addition to the unidirectional shift in bundle position, we encountered four cells that displayed biphasic hair-bundle motion following efferent stimulation (Fig. 4D).

To ascertain whether efferent activity modulates transient hair-bundle motion, we compared the steady-state bundle position 100 ms after a force pulse in the presence or absence of efferent stimulation. The mean bundle displacement in the positive direction after efferent activity was 94%±1% (mean ± SEM; ten cells, 76 pulses) of the control value (Fig. 4E). In contrast, the deflection during negative stimulation was 100%±1% of the control level (56 pulses analyzed). Similar results were obtained with physiological ionic conditions or with a single chamber containing saline solution with 1.5 mM Ca^2+^.

In summary, we found that efferent stimulation affects the motion of the hair bundle by shifting the bundle's position. The rapidity of this effect is consistent with a mechanism involving efferent modulation of the hair bundle's Ca^2+^ concentration through a change in the driving force on Ca^2+^, rather than diffusion of Ca^2+^ from intracellular stores to the hair bundle.

## Discussion

### Efferent innervation in the sacculus

We found that the efferent pathway differentially innervates distinct types of hair cells in the bullfrog's sacculus. The flask-shaped cells containing the Ca^2+^ buffer calretinin and expressing large Ca^2+^-dependent K^+^ currents receive a greater number of efferent terminals than the cylindrical cells with large voltage-dependent Ca^2+^ currents. This pattern accords with the high sensitivity of the flask-shaped cells to apamin [6], which reflects the presence of many efferent-activated SK channels. Although we generated polyclonal antibodies against anuran SK channels, their specificity was inadequate to reveal the spatial distribution of the channel in saccular hair cells. Together with the fact that calretinin-positive hair cells contain fewer afferent synaptic ribbons, our observation indicates that there are significant differences in the synaptic properties of the two types of saccular hair cells. Moreover, the greater efferent innervation of calretinin-positive cells, along with the total absence of synaptic ribbons in extramacular hair cells, suggests a role for calretinin-positive hair cells that may not be limited to afferent synaptic transmission. Although the extramacular squamous epithelium is not covered by the otolithic membrane, the kinocilia of extramacular hair cells could be influenced by the motion of the otoconial mass [39]. A better understanding of the micromechanical properties of extramacular hair bundles would be necessary to evaluate the significance of mechanical forces exerted by extramacular kinocilia following efferent stimulation.

In addition to the robust immunofluorescence signal for synapsin I in efferent terminals, a fainter but consistent signal was localized adjacent to synaptic ribbons when the gain of the confocal image was increased nearly to saturation (Fig. S1D). It is conceivable that the antibodies also recognize at the afferent synapses of hair cells expression of lower levels of synapsin I or of another isoform with a conserved epitope (Fig. S1A,B).

### Action potentials *versus* electrical resonance in hair cells

We encountered many saccular hair cells that generated action potentials in response to depolarization. Action potentials have been reported in hair cells from the sacculi of bullfrogs and leopard frogs [2], [29], the sacculus, lagena, and utriculus of goldfish [40], the semicircular canals of chicks [41], and the cochleas of turtles and alligators [42], [43], [44]. In mammalian and avian cochleas, action potentials in hair cells seem to be restricted to early developmental ages [45], [46].

In spite of the significant number of reports, the physiological relevance of action potentials in hair cells remains unknown. In principle, hair cells should lose their frequency-tuning properties by leaving a subthreshold regime. However, the generation of action potentials may not interfere with the tuning of those hair cells sensitive to frequencies below roughly 50 Hz, in which a spike could occur during the depolarizating phase of each cycle. In fact, this strategy would increase the synaptic efficiency for low-amplitude stimuli with no detriment to tuning. Alternatively, the sacculus might sacrifice frequency information for the sake of temporal resolution, thus becoming more sensitive to faint, transient stimuli.

### Effect of efferent activity on the hair cell's receptor potential

We have investigated efferent modulation of the hair cell's receptor potential in resonant as well as spiking saccular hair cells. Electrical stimulation of efferent axons evoked large inhibitory postsynaptic potentials that degraded hair-cell frequency tuning and suppressed firing.

In agreement with previous reports from turtle and mammals [23], [28], we found a two-component response in most saccular hair cells. The brief depolarizing component was not observed, however, in a previous report utilizing a similar preparation [21]. The inhibitory effect of efferent stimulation on the tuning properties of saccular hair cells accords with findings in the turtle's cochlea [47]. Consistent with a previous report from developing mammalian inner hair cells [23], efferent stimulation suppressed Ca^2+^ action potentials. The rare postinhibitory excitation recorded in some hair cells resembles that in intermediate-bouton afferents of the turtle's posterior ampullary nerve [30] and raises the possibility of an excitatory role for the efferent pathway.

### Efferent regulation of hair-bundle mechanics

We found that efferent stimulation shifts the resting position of a hair bundle by a few nanometers without a striking change in bundle stiffness. Both positive and negative motions were evoked by efferent stimulation. We observed these effects in the large, cylindrical, calretinin-negative, centrally located hair cells of the sacculus, whose mechanical properties are well established. Given that calretinin-positive cells are more densely innervated by efferent terminals, we cannot rule out a different efferent effect on these cells. Comparable directional variability has been described in the turtle's cochlea following intensely depolarizing current pulses [48]. Similarly, in the bullfrog's sacculus, the directionality of the movement evoked by a decrease of the extracellular Ca^2+^ concentration can be reversed by application of a positive mechanical offset, which presumably modifies the resting Ca^2+^ concentration within the stereocilia [49]. After the application of a displacement pulse, electrical stimulation of efferent axons shifted the resting point of the hair bundle several tens of nanometers in the positive direction. Diffusion in one dimension can be approximately described by the equation *x* ^2^ = 2*Dt*, in which *x* is the distance, *D* is the diffusion coefficient [50], and *t* is the time. Even in the absence of buffers, Ca^2+^ would require nearly 1 s to diffuse from the membrane postsynaptic to an efferent terminal to the hair bundle. This timescale indicates that the efferent action in our experiments is unlikely to stem from the diffusion of Ca^2+^ from efferent synapses to the site of adaptation. Our results are consistent, however, with an efferent effect on the hair bundle's Ca^2+^ concentration through an increase in the Ca^2+^ driving force. It is possible that efferent activation reduces hair-cell sensitivity by hampering the resetting of the hair bundle's position to its normal operating point. Because not all responses to mechanical pulses could be fitted satisfactorily with exponential functions, further experiments will be necessary to reveal a potential role of efferent activation on adaptation kinetics.

## Supporting Information

Figure S1 Characterization of anti-synapsin antiserum. A, In the top panel, an alignment of the N-termini of synapsin I in three different species shows the conserved sequence (underlined in the Xenopus laevis sequence) used for antiserum generation. NCBI accession numbers are in parentheses. The middle panel displays an alignment of the synapsin isoforms found in Xenopus laevis. The bottom panel shows an alignment of all synapsin isoforms found in the mouse Mus musculus. Blue denotes residues conserved among all sequences, red indicates residues present in two of the three sequences, and green shows residues conserved in two of the three isoforms. B, The right panel presents an immunoblot loaded with brain extract from the bullfrog, mouse, and chicken and incubated with the purified Rb1498 antiserum against synapsin I. Immunoreactive bands occur at approximately 80 kDa and 55 kDa. The lower bands might correspond to synapsin isoforms or degradation products. The left panel demonstrates that the presence of the peptide used for immunization eliminates immunoreactivity. C, A confocal section (lower panels) shows the presence of efferent terminals at the level of hair cells in the saccular macula. The characteristic punctate labeling by the anti-synapsin I antiserum is absent (upper panels) after preadsorption of the antiserum with the corresponding antigen. Both samples were assayed in parallel and Z-stack confocal sections were obtained with the identical acquisition settings. D, A maximal-intensity projection of confocal Z-stacks illustrates efferent terminals labeled by an anti-synapsin I purified antiserum (Gp118) (synapsin I, arrowheads) and synaptic ribbons (CtBP2) in the basolateral region of hair cells. Note the anti-synapsin I antibody faintly labels afferent postsynaptic terminals localized adjacent to synaptic ribbons. Scale bars: C, 10 µm; D, 5 µm.
(3.54 MB TIF)

Figure S2 A, Applying a total of 120 consecutive single stimuli to efferent fibers elicited 73 inhibitory postsynaptic potentials of a wide variety of magnitudes from a hair cell maintained in 4 mM Ca2+. B, The amplitude and area of the inhibitory postsynaptic potentials recorded in another cell are plotted against the delay between pairs of efferent shocks (mean ± SEM; number of events: N2 ms  = 27, N3 ms  = 39, N5 ms  = 120, N10 ms  = 36, N30 ms  = 42, N40 ms  = 37). C, Injection of depolarizing current pulses (lower traces) triggered action potentials in a hair cell. The amplitude and frequency of the spikes depended on the level of depolarization. The cell was maintained in a two-compartment chamber with 4 mM Ca2+ endolymph and 2 mM Ca2+ standard saline solution. D, Single action potentials occurred both at the onset of depolarization and as rebound spikes following hyperpolarizing current steps. The cell was exposed to 2 mM Ca2+ standard saline solution. E, Efferent stimulation (asterisk) inhibited spontaneous oscillatory activity at the resting potential. F, An excitatory efferent effect was occasionally obtained after the hyperpolarizing component of the inhibitory postsynaptic potential. The records show the responses to four consecutive efferent shocks from a hair cell bathed in 4 mM Ca2+ standard saline solution. Notice the failure of spike generation on one occasion (asterisk). The inset shows action potentials recorded in the same cell after depolarization as well as rebound spikes following hyperpolarizing current steps (scale bar: 100 ms).
(2.60 MB TIF)

Figure S3 Effect of efferent stimulation on the motion of a bundle. A, A hair bundle was mechanically stimulated by ±50 nm at 60 Hz with a flexible glass fiber (middle trace). Following efferent stimulation (bottom trace), the bundle's movement (upper trace) was significantly reduced. B, When another hair bundle was subjected to the same paradigm with 30 Hz stimulation of ±10 nm, its motion was augmented after efferent stimulation. Each trace is the average of 20–30 repetitions recorded in a two-compartment preparation with 0.25 mM Ca2+ endolymph and 2 mM Ca2+ standard saline solution. Upward deflections denote movement in the positive direction, towards the kinocilium.
(2.35 MB TIF)

Video S1 A confocal Z-stack animation of the macular periphery illustrates the presence of efferent terminals exclusively at the basolateral region of hair cells. Synapsin I is shown in red, parvalbumin 3 in green, and DAPI-labeled nuclei in blue. The beginning of the movie corresponds to the somata of the supporting cells and the end to the apical surfaces and hair bundles of the hair cells.
(15.52 MB AVI)

Video S2 A three-dimensional animation of a confocal stack of images of two extramacular hair cells depicts the presence of efferent terminals contacting their basolateral region. Synapsin I is shown in red and calretinin in green.
(0.69 MB AVI)

Video S3 A confocal Z-stack animation of the macula illustrates the occurrence of synaptic ribbons of different sizes labeled by an antiserum against CtBP2 (green). Nuclei are labeled with DAPI (blue). The movie progresses from the apical surfaces of hair cells, which are faintly marked by anti-CtBP2, to the nuclear layer of supporting cells. Note the presence of a mitotic figure in the lower left corner.
(4.93 MB AVI)
