# Supplementary figures and images for: Efferent Control of the Electrical and Mechanical Properties of Hair Cells in the Bullfrog's Sacculus
Source: PLoS One. 2010 Oct 29;5(10):e13777. doi: 10.1371/journal.pone.0013777 (PMC2966443; doi:10.1371/journal.pone.0013777)

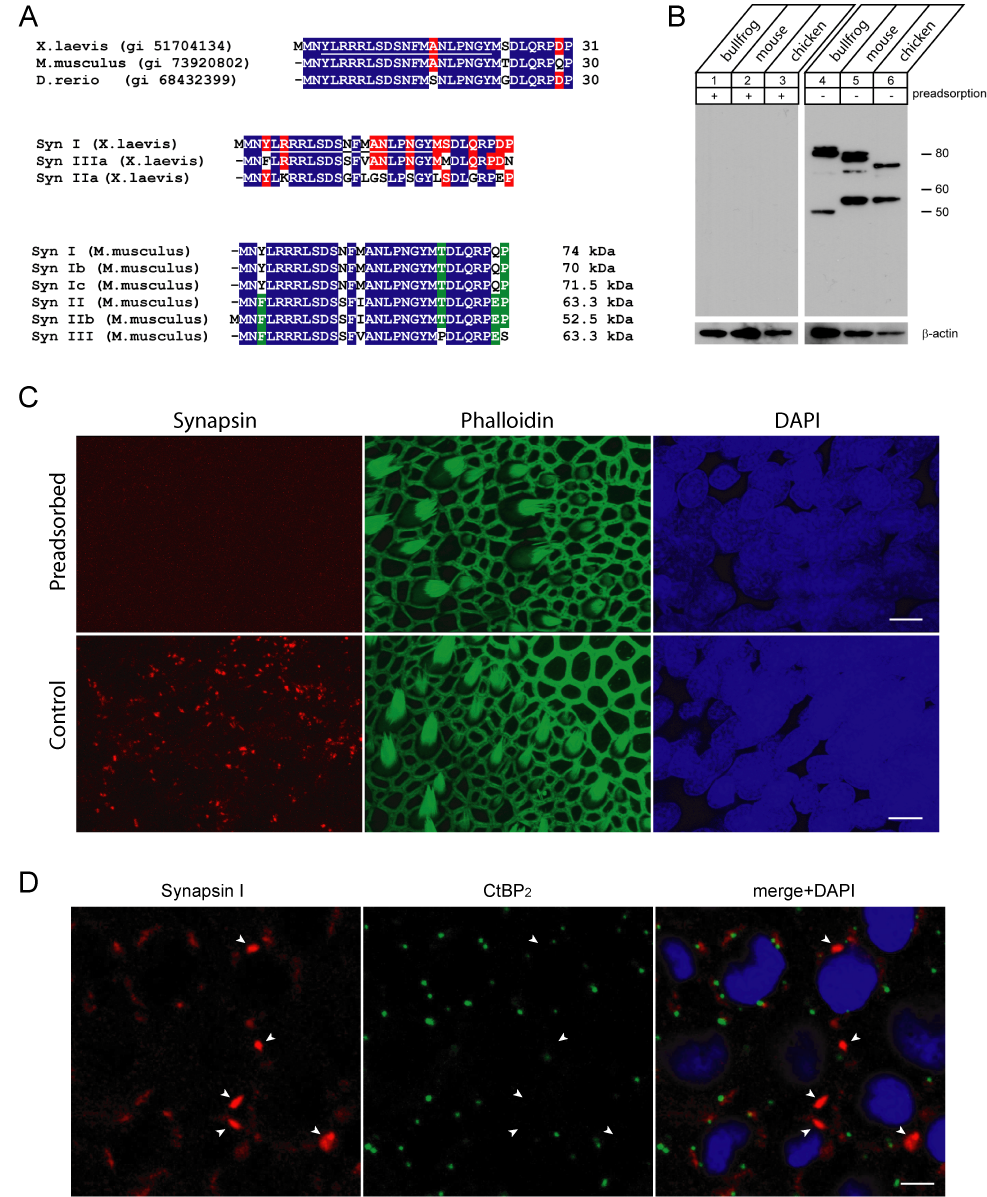

Supplement: Figure S1 — Characterization of anti-synapsin antiserum. A, In the top panel, an alignment of the N-termini of synapsin I in three different species shows the conserved sequence (underlined in the Xenopus laevis sequence) used for antiserum generation. NCBI accession numbers are in parentheses. The middle panel displays an alignment of the synapsin isoforms found in Xenopus laevis. The bottom panel shows an alignment of all synapsin isoforms found in the mouse Mus musculus. Blue denotes residues conserved among all sequences, red indicates residues present in two of the three sequences, and green shows residues conserved in two of the three isoforms. B, The right panel presents an immunoblot loaded with brain extract from the bullfrog, mouse, and chicken and incubated with the purified Rb1498 antiserum against synapsin I. Immunoreactive bands occur at approximately 80 kDa and 55 kDa. The lower bands might correspond to synapsin isoforms or degradation products. The left panel demonstrates that the presence of the peptide used for immunization eliminates immunoreactivity. C, A confocal section (lower panels) shows the presence of efferent terminals at the level of hair cells in the saccular macula. The characteristic punctate labeling by the anti-synapsin I antiserum is absent (upper panels) after preadsorption of the antiserum with the corresponding antigen. Both samples were assayed in parallel and Z-stack confocal sections were obtained with the identical acquisition settings. D, A maximal-intensity projection of confocal Z-stacks illustrates efferent terminals labeled by an anti-synapsin I purified antiserum (Gp118) (synapsin I, arrowheads) and synaptic ribbons (CtBP2) in the basolateral region of hair cells. Note the anti-synapsin I antibody faintly labels afferent postsynaptic terminals localized adjacent to synaptic ribbons. Scale bars: C, 10 µm; D, 5 µm. (3.54 MB TIF) [file pone.0013777.s001.tif]

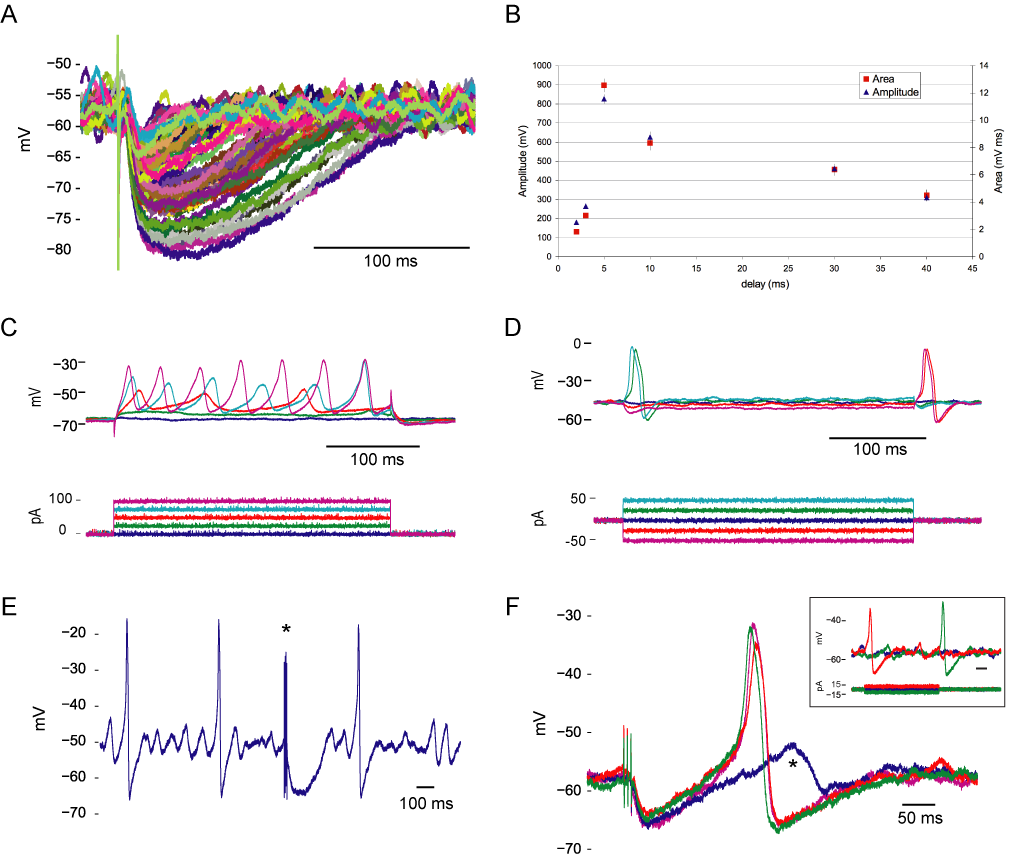

Supplement: Figure S2 — A, Applying a total of 120 consecutive single stimuli to efferent fibers elicited 73 inhibitory postsynaptic potentials of a wide variety of magnitudes from a hair cell maintained in 4 mM Ca2+. B, The amplitude and area of the inhibitory postsynaptic potentials recorded in another cell are plotted against the delay between pairs of efferent shocks (mean ± SEM; number of events: N2 ms = 27, N3 ms = 39, N5 ms = 120, N10 ms = 36, N30 ms = 42, N40 ms = 37). C, Injection of depolarizing current pulses (lower traces) triggered action potentials in a hair cell. The amplitude and frequency of the spikes depended on the level of depolarization. The cell was maintained in a two-compartment chamber with 4 mM Ca2+ endolymph and 2 mM Ca2+ standard saline solution. D, Single action potentials occurred both at the onset of depolarization and as rebound spikes following hyperpolarizing current steps. The cell was exposed to 2 mM Ca2+ standard saline solution. E, Efferent stimulation (asterisk) inhibited spontaneous oscillatory activity at the resting potential. F, An excitatory efferent effect was occasionally obtained after the hyperpolarizing component of the inhibitory postsynaptic potential. The records show the responses to four consecutive efferent shocks from a hair cell bathed in 4 mM Ca2+ standard saline solution. Notice the failure of spike generation on one occasion (asterisk). The inset shows action potentials recorded in the same cell after depolarization as well as rebound spikes following hyperpolarizing current steps (scale bar: 100 ms). (2.60 MB TIF) [file pone.0013777.s002.tif]

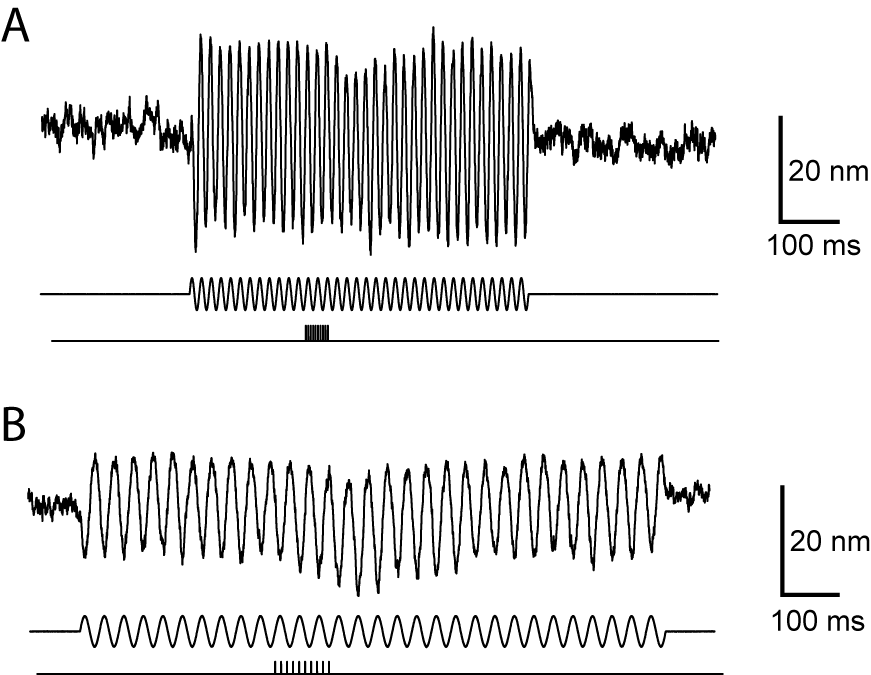

Supplement: Figure S3 — Effect of efferent stimulation on the motion of a bundle. A, A hair bundle was mechanically stimulated by ±50 nm at 60 Hz with a flexible glass fiber (middle trace). Following efferent stimulation (bottom trace), the bundle's movement (upper trace) was significantly reduced. B, When another hair bundle was subjected to the same paradigm with 30 Hz stimulation of ±10 nm, its motion was augmented after efferent stimulation. Each trace is the average of 20–30 repetitions recorded in a two-compartment preparation with 0.25 mM Ca2+ endolymph and 2 mM Ca2+ standard saline solution. Upward deflections denote movement in the positive direction, towards the kinocilium. (2.35 MB TIF) [file pone.0013777.s003.tif]
